# 

**Published:** 1915-07

**Authors:** 


### CONTENTS.

ORIGINAL COMMUNICATIONS—

Pathology of Tuberculosis, by E. C. Thrash, M. D., Atlanta, Ga. .......... 159
Subjective and Objective Symptomatology of Tuberculosis, by Louis C. Roughlin, M. D. .......... 164
Auscultation in Pulmonary Tuberculosis, by Arch Elkin, M. D. .......... 168
Treatment of Pulmonary Tuberculosis, by E. C. Thrash, M. D. .......... 170
The Symptom Flatulence and its Symptomatic Treatment, By George M. Niles, M. D. .......... 174
The Roll of Emetine in the Treatment of Pyorrhea Alveolaris, by William Crenshaw, D. D. S., Dean, Atlanta Dental College .......... 179
Regular Meeting of the Fulton County Medical Society, July 1, 1915, by Allen H. Bunce, M. D. .......... 182
EDITORIAL .......... 188
SELECTIONS AND ABSTRACTS .......... 190
BOOK REVIEWS .......... 205

